# Transgenic mice lacking CREB and CREM in noradrenergic and serotonergic neurons respond differently to common antidepressants on tail suspension test

**DOI:** 10.1038/s41598-017-14069-6

**Published:** 2017-10-18

**Authors:** Katarzyna Rafa–Zabłocka, Grzegorz Kreiner, Monika Bagińska, Justyna Kuśmierczyk, Rosanna Parlato, Irena Nalepa

**Affiliations:** 1 Department Brain Biochemistry, Institute of Pharmacology, Polish Academy of Sciences, 31-343 Krakow, Smetna 12, Poland; 2 Institute of Applied Physiology, University of Ulm, 89081 Ulm, Germany; 3 Institute of Anatomy and Cell Biology, University of Heidelberg, 69120 Heidelberg, Germany

## Abstract

Evidence exists that chronic antidepressant therapy enhances CREB levels and activity. Nevertheless, the data are not conclusive, as previous analysis of transgenic mouse models has suggested that CREB inactivation in fact contributes to antidepressant-like behavior. The aim of this study was to evaluate the role of CREB in this context by exploiting novel transgenic mouse models, characterized by selective ablation of CREB restricted to noradrenergic (Creb1^DBHCre^/Crem−/−) or serotonergic (Creb1^TPH2CreERT2^/Crem−/−) neurons in a CREM-deficient background to avoid possible compensatory effects of CREM. Selective and functional ablation of CREB affected antidepressant-like behavior in a tail suspension test (TST) after antidepressant treatment. Contrary to single Creb1^DBHCre^ mutants, Creb1^DBHCre^/Crem−/− mice did not respond to acute desipramine administration (20 mg/kg) on the TST. On the other hand, single Creb1^TPH2CreERT2^ mutants displayed reduced responses to fluoxetine (10 mg/kg) on the TST, while the effects in Creb1^TPH2CreERT2^/Crem−/− mice differed by gender. Our results provide further evidence for the important role of CREM as a compensatory factor. Additionally, the results indicate that new models based on the functional ablation of CREB in select neuronal populations may represent a valuable tool for investigating the role of CREB in the mechanism of antidepressant therapy.

## Introduction

Depression is a mental illness affecting complex cognitive and emotional functions with increasing prevalence in modern, highly industrialized societies. Decreased monoamine levels in the central nervous system represent a crucial element of the monoamine hypothesis of depression that currently dominates our understanding of the pathophysiological basis of this illness and its pharmacological treatment^1,2^. Even though there are serious limitations to the monoamine theory as it does not provide a complete explanation regarding neither the mechanism of action of antidepressants, nor the basis of depression pathophysiology, it is supported by the fact that antidepressants that enhance the levels of two important neurotransmitters in the brain, noradrenaline and serotonin, alleviate depression symptoms^3^. Additional mechanisms contributing to the pathology of depression include dysfunction of the hypothalamic–pituitary–adrenal (HPA) axis, inflammatory alterations, and epigenetic mechanisms^2^. Nevertheless, the treatment of choice for depression is primarily based on the modulation of noradrenergic or/and serotonergic signal transmission.

There have been several attempts to identify a protein that could serve as a convergence point for antidepressant treatment, the most prominent example being the cyclic AMP response element binding protein (CREB) transcription factor^4^, one of transcription factors binding to cAMP-responsive elements (CRE). CREB appears to be involved in both the mechanisms of antidepressant action and in the disease itself ^5^. Therefore, research has focused on the role of G-protein-coupled receptors and associated second messenger pathways, primarily involving the cyclic AMP (cAMP)-dependent pathway. Augmentation of cAMP leads to the activation of cAMP-dependent protein kinase A (PKA), and PKA activity is enhanced after chronic antidepressant treatment^6,7^. Alternative mechanisms implicated in the action of antidepressant drugs are related to Ca2±/calmodulin-dependent protein kinase II (CaMKII)^8,9^. CREB has been demonstrated to mediate the transcriptional activation of genes in response to both cAMP and Ca^2+^ influx signal transduction pathways^10,11^. For instance, according to the neurotrophic hypothesis of depression, levels of brain derived neurotrophic factor (BDNF, whose transcription is regulated by a CREB-dependent mechanism) are increased both by noradrenergic or/and serotonergic antidepressants, while exposure to stress is characterized by downregulation of BDNF expression in hippocampus^12^; this downregulation can be prevented by antidepressant treatment^13,14^. However, it has to be mentioned, that this effect is not specific and although enhancement of BDNF expression seems to exert antidepressant-like effects in the hippocampus, its actions might be opposite in other brain regions i.e. nucleus accumbens, where chronic social defeat stress increases BDNF protein levels^15^. BDNF also regulates serotonin signaling, as its main receptor, TrkB, can be found on serotonergic neurons as well^16^.

The abovementioned monoamine systems exert mutual influence over each other. The serotonergic system may be inhibited by noradrenaline through action on α1- and β-adrenergic receptors on serotonergic neurons of the raphe nuclei, while serotonergic projections can inhibit the activity of the locus coeruleus^6,17^.

Antidepressants can affect CREB in several different ways; however, the data are not conclusive. The generally accepted theory is that chronic antidepressant treatment enhances CREB levels and activity, thus implicating CREB as an important mechanism of antidepressant treatment^5^. In particular, chronic administration of desipramine and imipramine (common antidepressants whose action is based primarily on the inhibition of noradrenaline reuptake) increases expression of CREB mRNA and phospho-CREB (pCREB) in selected brain regions^18,19^. Similar effects have been observed after fluoxetine or citalopram (selective serotonin reuptake inhibitors, or SSRIs) treatment^18,20^. On the other hand, adverse effects on CREB expression have also been observed after desipramine or fluoxetine treatment^21,22^. Furthermore, treatment with venlafaxine, a dual monoamine reuptake inhibitor, was shown to reduce pCREB in frontal cortex with no change in the total CREB expression^23^.

Rodent models have made substantial contributions to advancing our understanding of depression and the mechanism of antidepressant treatment. Several models for studying the role of CREB are based on transgenic rats overexpressing CREB^24,25^ or mice with a constitutive deletion of the gene^26–28^. Results from these models are counterintuitive, as the majority of studies have demonstrated that CREB inactivation contributes to antidepressant-like behavior. However, it should be emphasized that these loss-of-function studies possess many caveats, possibly making interpretation of the data difficult and misleading. Namely, (i) the experimental mutations generally targeted CREB in several brain structures; and (ii) compensatory effects of related CREB heterodimerization gene products (i.e., cAMP response element modulator, CREM) were not taken into consideration. The last caveat seems to be of particular importance, as it was shown that CREB is not the only mediator of cAMP-dependent transcriptional regulation and other nuclear effectors of the cAMP-dependent signaling pathway can compensate lack of CREB function^29^. In particular, CREB spatiotemporal knockouts are usually not as deteriorated regarding their phenotype as expected, as other c-AMP driven transcriptional activators (CREM and ATF-1) can compensate for each other, and various CREB deficient mice overexpress CREM^29^. This strong interdependence between CREB and CREM was also confirmed in the study of Mantamadiotis *et al*., showing that the embryonic mutation evoking loss of CREB in neural and glial progenitors (Creb1^NesCre^Crem−/− mice) was effective throughout the brains of these mutants only when both CREM alleles were also depleted^30^.

The aim of the current study was to evaluate the function of CREB in the mechanisms of antidepressant treatment by exploiting novel transgenic mouse models characterized by selective functional ablation of CREB restricted only to the noradrenergic or serotonergic neurons. Furthermore, considering the known compensatory effects of CREM^29,31^, both lines were maintained in a CREM-deficient (Crem−/−) background.

## Materials and Methods

### Animals

Selective ablation of CREB in noradrenergic and serotonergic systems (Creb1^DBHCre^ and Creb1^TPH2Cre^ mice, respectively) was achieved by *Cre/loxP* recombination system. Transgenic mice (C57Bl/6N background) hosting *Cre* recombinase under the dopamine beta-hydroxylase (DBH) promoter (DBHCre mice)^32^ or tryptophan hydroxylase 2 (TPH2) promoter (TPH2Cre mice)^33^ were crossed with animals harboring the floxed Creb1 gene. The TPH2Cre line was created in inducible form (TPH2CreERT2)^33^. Induction of the inducible Cre recombinase was achieved by injection of 2 mg of tamoxifen (Sigma-Aldrich, USA) at least 4 weeks prior to any experimental procedure (once daily, i.p., for 5 consecutive days; tamoxifen dissolved in a 10:1 oil:ethanol mixture). Both lines were kept in a CREM-deficient (Crem−/−) background, as described previously^30^. We thus obtained two transgenic lines with functional deletion of CREB restricted to noradrenergic (Creb1^DBHCre^/Crem−/−) or serotonergic (Creb1^TPH2CreERT2^/Crem−/−) neurons. Genotyping was performed with a commercially available kit (AccuStart™ II Mouse Genotyping Kit, QuantaBio/VWR) according to the manufacturer’s protocol using the following primers: DBHCre: forward 5′-CTG CCA GGG ACA TGG CCA GG-3′, reverse 5′-GCA CAG TCG AGG CTG ATC AGC-3′; TPH2Cre: forward 5′ TGC AAC GAG TGA GG TTC-3′, reverse 5′-ATG TTT AGC TGG CCC AAA TG 3′; Creb1flox: forward 5′-TAT GTA AAG CAA GGG AAG ATA CTG-3′, reverse 5′-TAG ACA TAC TTG ACC CAT AGC ATT-3′; and CREM knockout: forward 5′-TGG ATT GTG CTG GGA GGT TGT TC-3′, reverse 5′-TCT TTG AGG GCC TTG AGT TCC TC-3′).

Male and female mutant mice were kept with their control (Cre-negative, CREM+/+) littermates of the same sex in self-ventilated cages under standard laboratory conditions (12 h light/dark cycle, food and water ad libitum). All mice were 3–4 months old (approx. 12–16 weeks). This study was carried out in strict accordance with the recommendations of the Guide for the Care and Use of Laboratory Animals of the National Institutes of Health. Behavioral protocols were approved by the Animal Ethical Committee at the Institute of Pharmacology, Polish Academy of Sciences (Permit Number: 1125, issued 11/24/2014).

### Drugs

Desipramine (20 mg/kg, i.p., Sigma-Aldrich, USA) and fluoxetine (10 mg/kg, i.p., Carbosynth, U.K.) were injected 30 min prior to the test. The control groups received 0.9% NaCl.

### Immunofluorescence

Mice were sacrificed by cervical dislocation. Their brains were removed and fixed in 4% paraformaldehyde (PFA) overnight. After dehydration, the tissue was embedded in paraffin and coronally sectioned (7 µm) on a rotary microtome (Leica, RM45). Select sections from the corresponding region of the locus ceruleus (LC), dorsal raphe nuclei (DR), hippocampus, or frontal cortex in mutant and control mice were incubated overnight at 4 °C with primary anti-CREB (1:100, Abcam, United Kingdom, cat no. ab32515), anti-NeuN (1:100, Millipore, USA, cat. no. MAB377), anti-Tph2 (1:100, Millipore, USA, cat. no. AB1541) and anti-TH (1:500, Millipore, USA, cat. no. AB1542) antibodies. Antigen-bound primary antibodies were visualized with anti-rabbit Alexa-488, anti-sheep Alexa-594, and anti-mouse Alexa-594 (Invitrogen, USA) coupled secondary antibodies. Stained sections were analyzed and acquired under a fluorescence microscope (Nikon Eclipse50i, Japan) equipped with a camera and specialized software (NIS Elements, ver. BR 3.0).

### Open field test (OFT)

The OFT was performed to assess spontaneous locomotor activity. Mice were tracked by video camera using automated video tracking software (EthoVision XT8, Noldus, Netherlands) for 60 min in 40 × 40 cm white square boxes; the total distance moved was scored in 10-min intervals.

### Rotarod test (ROT)

The ROT was performed to assess motor coordination using an accelerated rotarod (Ugo Basile, Italy). The assessment was preceded by a training session 1 day before the experiment (5 min on the rotating rod, constant speed of 4 rpm). During the experiment the time spent on the accelerating rod (4–40 rpm in a 5-min period) was measured.

### Tail suspension test (TST)

The TST was performed to evaluate depression-like and antidepressant behavior after drug treatment. We recorded the overall time that animals were immobile while suspended by the tail over a 6 min period. Scoring of immobility time was performed by means of automated video tracking software (EthoVision XT8, Noldus, Netherlands) as described previously^34^.

### Statistical analysis

Data were analyzed using Statistica 12 software (Statsoft, USA). All comparisons were performed using one-way analysis of variance followed by Fisher Least Significant Difference post-hoc test. Changes with p value lower than 0.05 were considered significant.

### Data Availability Statement

The datasets generated during and/or analyzed during the current study are available from the corresponding author on reasonable request.

## Results

### Ablation of CREB is cell type-specific in both studied transgenic lines

**Figure 1:**
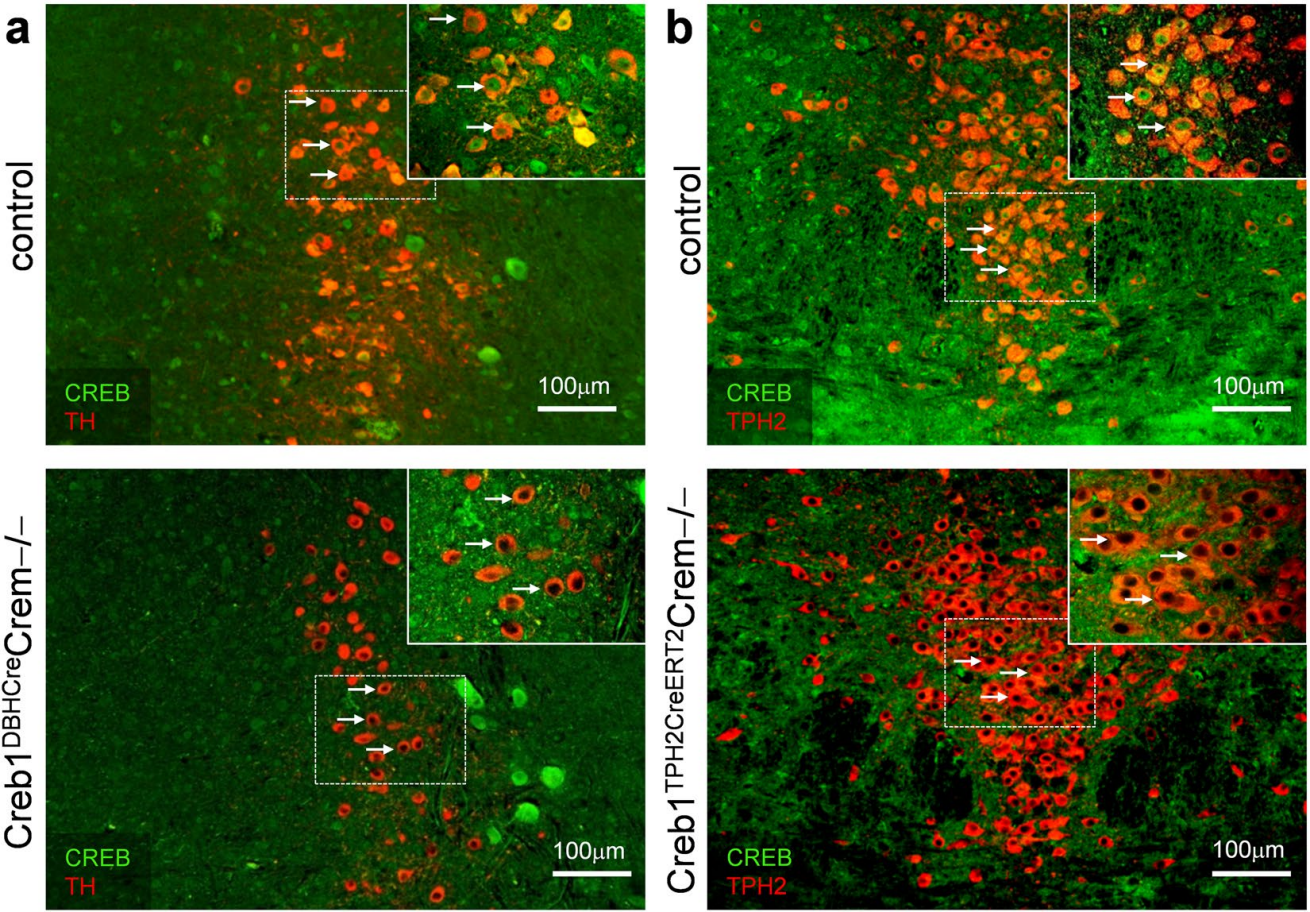
Immunofluorescent staining confirms cell type-specific deletion of CREB in noradrenergic and serotonergic neurons. (**a**) CREB (green) and TH (red) staining in locus coeruleus of control and Creb1^DBHCre^/Crem −/− mutant mouse. (**b**) CREB (green) and Tph2 (red) staining in dorsal raphe nucleus of control and Creb1^TPH2CreERT2^/Crem−/− mutant mouse. Scale bars: 100 µm.

**Figure 2:**
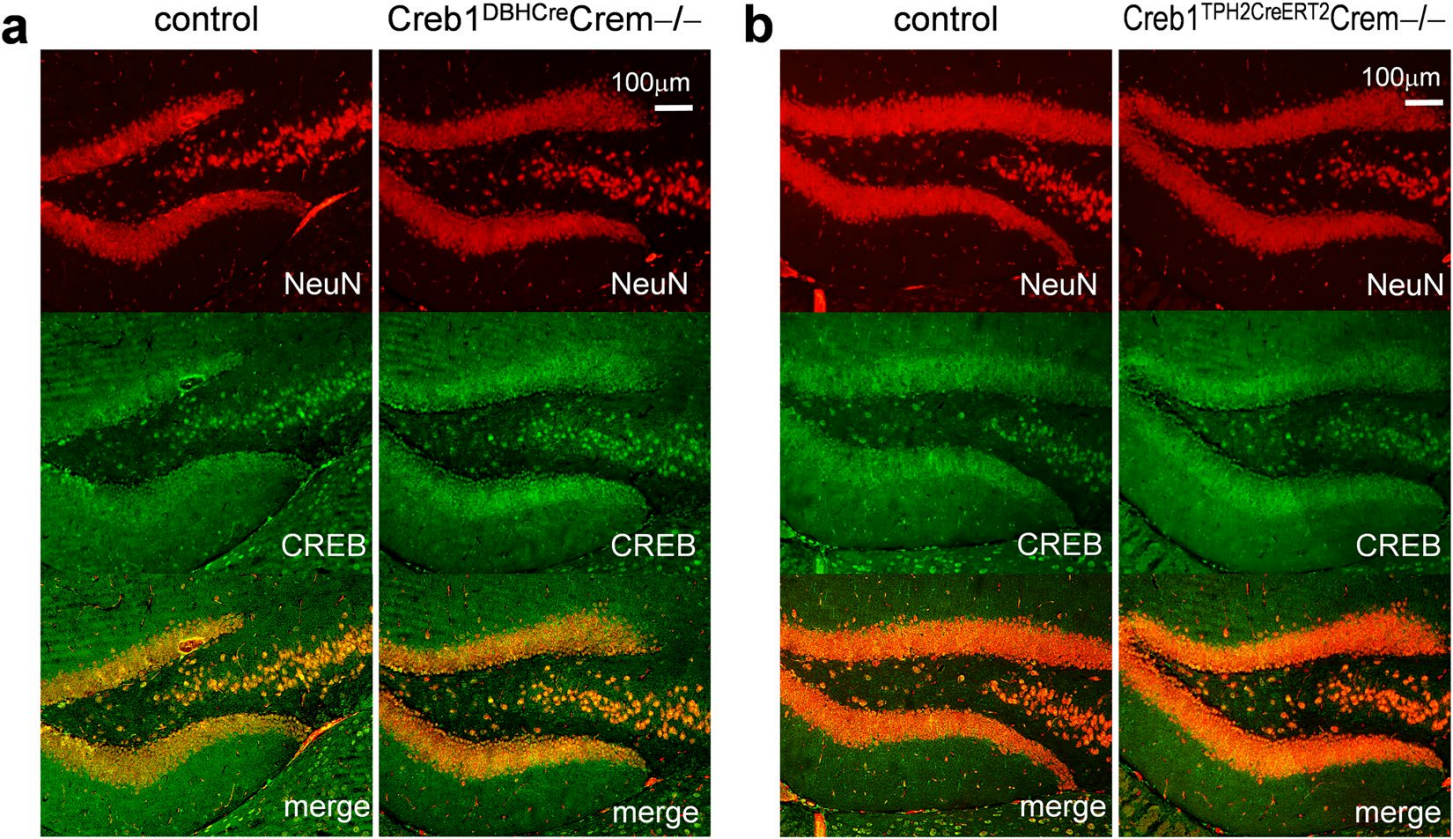
Immunofluorescent staining of hippocampal sections confirms promotor dependent selectivity of CREB deletion. CREB expressing cells are still present in the hippocampus – a brain structure not targeted by the mutation; (**a**) CREB (green) and NeuN (red) staining in the hippocampus of control and Creb1^DBHCre^/Crem −/− mutant mouse, (**b**) CREB (green) and NeuN (red) staining in the hippocampus of control and Creb1^TPH2CreERT2^/Crem−/− mutant mouse.

Immunofluorescent staining confirmed that the CREB protein was lost specifically in noradrenergic cells expressing tyrosine hydroxylase (TH) in the locus coeruleus (LC) of Creb1^DBHCre^Crem−/− mice (Fig. 1a) as well as in serotonergic, tryptophan hydroxylase 2 (TPH2) positive cells in the dorsal raphe (DR) nucleus of Creb1^TPH2CreERT2^Crem−/− mice (Fig. 1b). The DBHCre and the TPH2CreERT2 specificity have been previously shown and extensively used for conditional gene targeting. Here, we have shown that in both Creb1^DBHCre^Crem−/− and Creb1^TPH2CreERT2^Crem−/− lines, staining with anti-CREB antibodies provided no signal in the abovementioned brain structures of mutant animals. In other brain areas not targeted by the mutation (e.g., hippocampus), the CREB protein was preserved in both transgenic lines (Fig. 2a,b). These results confirmed that the mutations were highly specific in the central nervous system and restricted to noradrenergic and serotonergic neuronal cell populations.

### Male and female CREB- and CREB/CREM-deficient mice in serotonergic or noradrenergic cells show no impairment in basal behavioral phenotype

**Figure 3:**
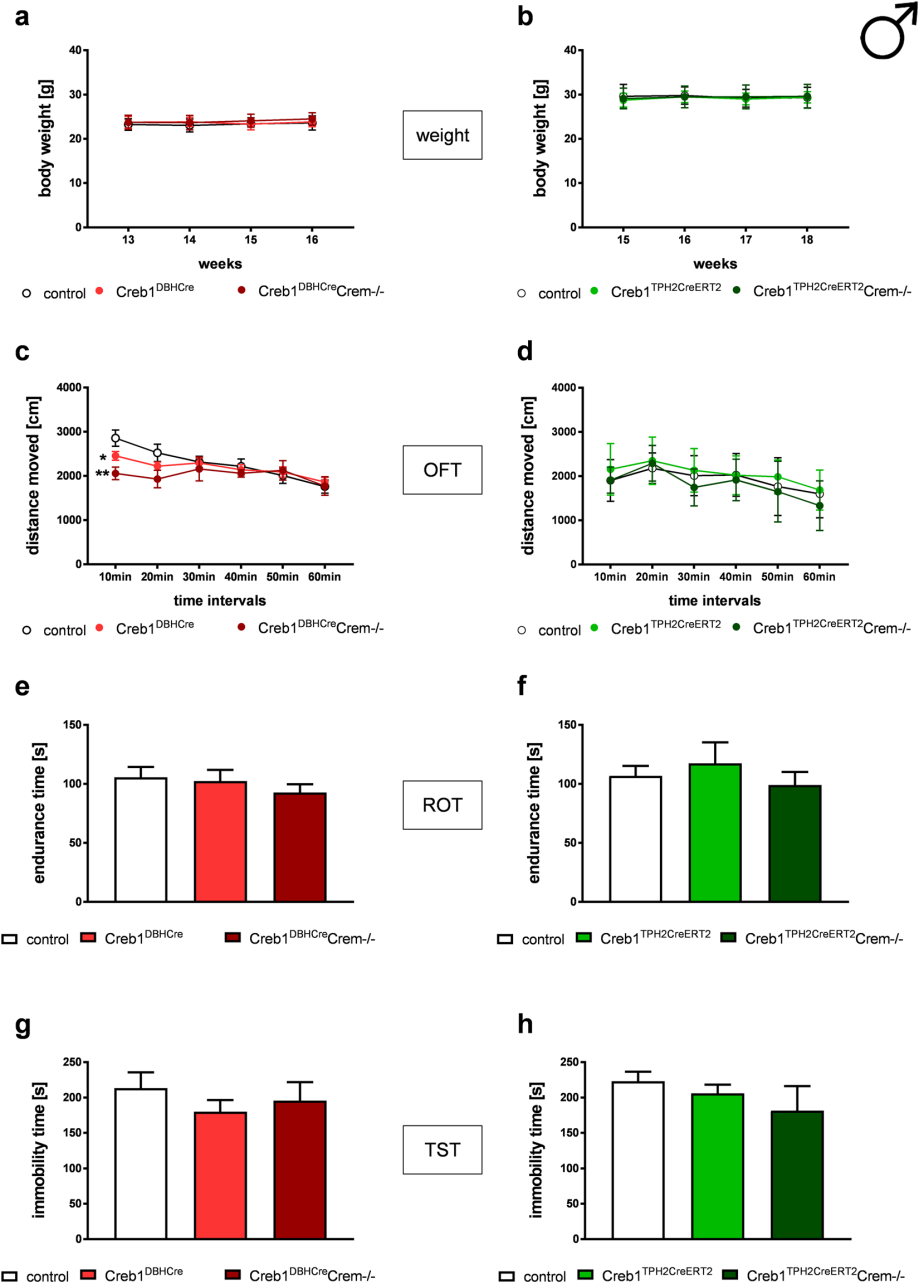
Male Creb1^DBHCre^/Crem−/− (left panel) and Creb1^TPH2Cre^/Crem−/− (right panel) mice do not show gross abnormalities and altered behavior under basal conditions. (**a**,**b**) body weight gain, (**c**,**d**) spontaneous locomotor activity assessed by the open field test (OFT) (**e**,**f**) motor coordination assessed by rotarod test (ROT), (**g**,**h**) depressive-like behavior assessed by tail suspension test (TST). Data are the mean ± SEM, n = 5–12. ANOVA: F_(2,29)_ = 6,035, p < 0.01; LSD post-hoc: *p < 0.05 Creb1^DBHCre^ vs control, **p < 0.01 Creb1^DBHCre^Crem−/− vs control mice (first 0–10 min interval, Fig. 3c).

Male Creb1^DBHCre^Crem−/− and Creb1^TPH2CreERT2^Crem−/− mice did not exhibit any visible impairments, nor was their body weight affected in comparison to their control littermates (Fig. 3a,b). Moreover, their spontaneous locomotor activity on the open field test (OFT) did not differentiate them from control animals, although in the first 10 min of the test, Creb1^DBHCre^Crem−/− mice were slightly less active (Fig. 3c,d). The ROT results demonstrated that the motor coordination of males in both transgenic mouse lines was unaffected by the mutation (Fig. 2e,f). Moreover, the TST did not reveal any differences in immobility times for single mutant Creb1^DBHCre^Crem−/− and double mutant Creb1^TPH2CreERT2^Crem−/− mice, suggesting the lack of a depressive or antidepressant phenotype under basal conditions (Fig. 3g,h).

**Figure 4:**
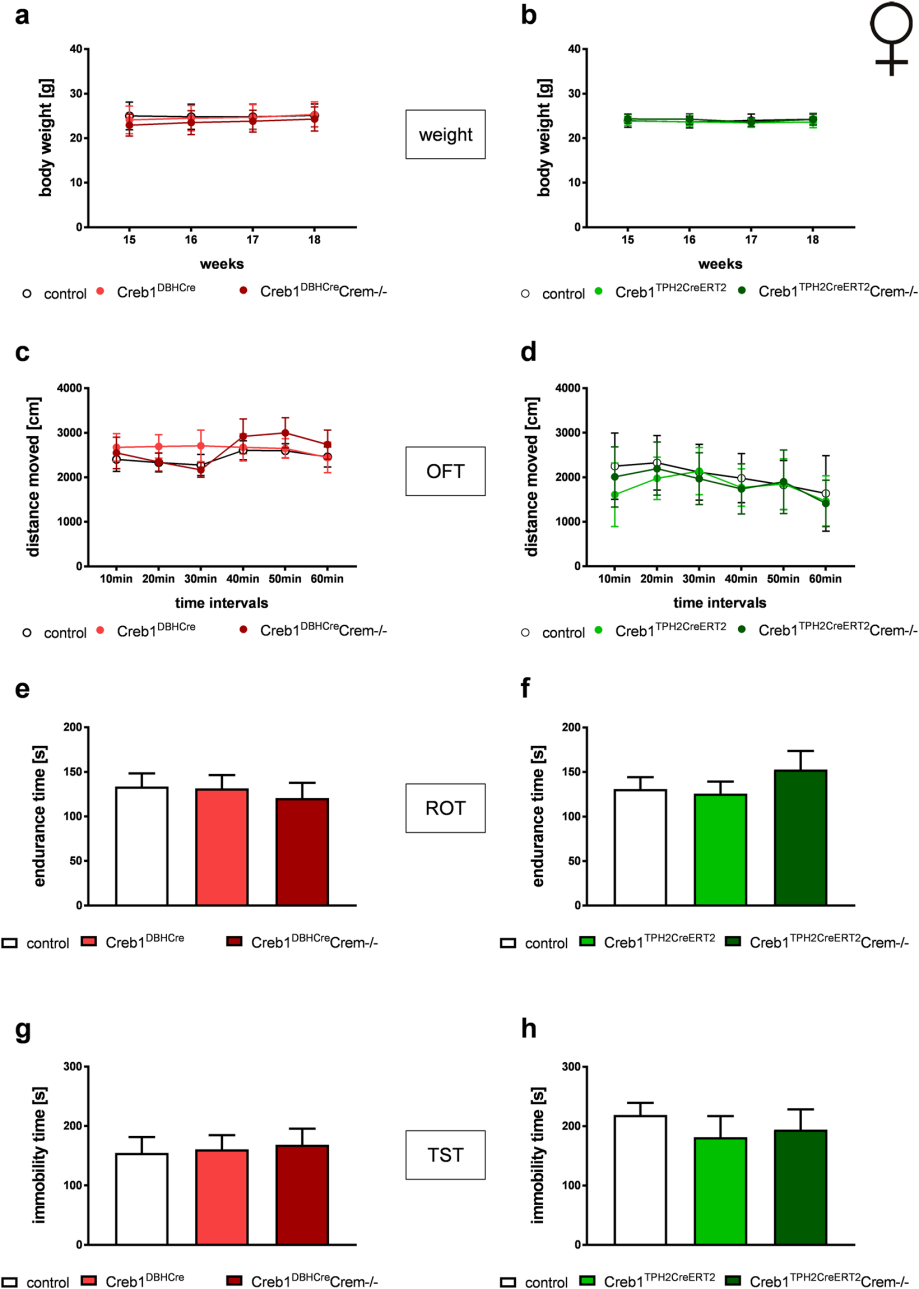
Female Creb1^DBHCre^/Crem−/− (left panel) and Creb1^TPH2Cre^/Crem−/− (right panel) mice do not show gross abnormalities and altered behavior under basal conditions. (**a**,**b**) body weight gain, (**c**,**d**) spontaneous locomotor activity assessed by open field test (OFT), (**e**, **f**) motor coordination assessed by rotarod test (ROT), (**g**,**h**) depressive-like behavior assessed by tail suspension test (TST). Data are the mean ± SEM, n = 5–12.

Similar to the mutant males, no differences were observed in the body weights of transgenic females of either the Creb1^DBHCre^Crem−/− or Creb1^TPH2CreERT2^Crem−/− line (Fig. 4a,b). As measured by the OFT and ROT, spontaneous locomotor activity (Fig. 4c,d) and motor coordination of female mutant mice (Fig. 4e,f) remained at similar levels as detected in control littermates, irrespective of the loss of CREB in noradrenergic and serotonergic neurons. Additionally, mutant females, like males, were indistinguishable from control mice in their TST response under basal conditions (Fig. 4g,h).

Overall, behavioral screening of basal phenotypes did not reveal any differences between control, single mutant (either Creb1^DBHCre^ or Creb1^TPH2Cre^) and double mutant (Creb1^DBHCre^Crem−/− or Creb1^TPH2CreERT2^Crem−/−) mice. These results indicated that neither of the introduced mutations affect the general health or basal behavior of the studied animals, regardless of sex.

### Creb1^DBHCre^Crem−/− double mutant mice, but not single Creb1^DBHCre^ mutants, are resistant to the antidepressant-like effects of desipramine treatment on the TST

**Figure 5:**
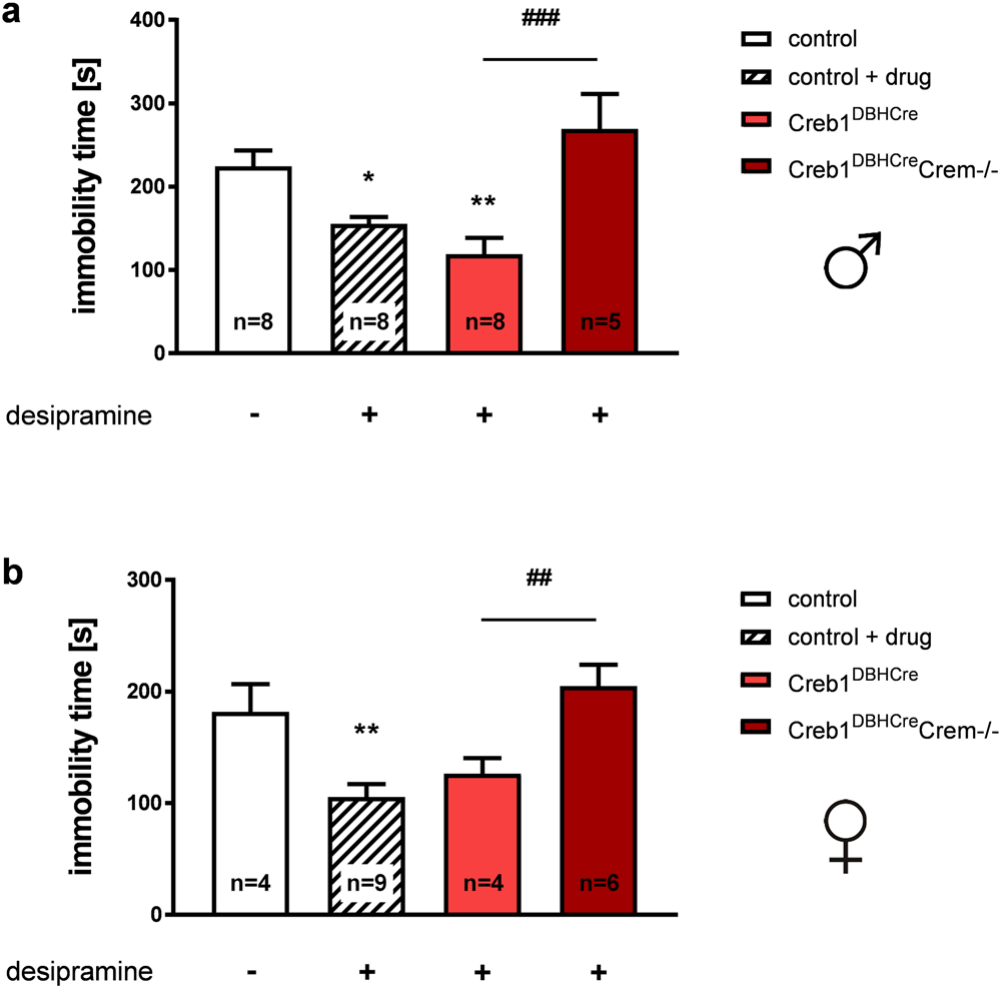
The effect of Creb1 deletion on mouse responsiveness on the TST after desipramine. (**a**) Immobility of control, Creb1^DBHCre^, and Creb1^DBHCre^/Crem−/− male mice on the TST after desipramine treatment (single dose, 20 mg/kg, i.p., 30 min. prior to the test) (ANOVA: F_(3,25)_ = 9.25, p < 0.001; LSD post-hoc: *p < 0.05, **p < 0.01 vs control; ^###^p < 0.001 vs Creb1^DBHCre^). (**b**) Immobility of control, Creb1^DBHCre^, and Creb1^DBHCre^/Crem−/− female mice on the TST after desipramine treatment (single dose, 20 mg/kg, i.p., 30 min prior to the test) (ANOVA: F_(3,19)_ = 8.71 p < 0.001; LSD post-hoc: **p < 0.01 vs control; ^##^p < 0.01 vs Creb1^DBHCre^). Data are the mean ± SEM, n = 4–9.

As expected, desipramine administration in control mice (both males and females) evoked an antidepressant behavior on the TST; this response is shown by their shortened immobility time in comparison to saline treated animals (Fig. 5a,b, second bars from the left). However, while single Creb1^DBHCre^ mutants showed an antidepressant desipramine response of similar level to control animals (significant only in males), Creb1^DBHCre^Crem−/− double mutants did not respond (Fig. 5a,b), thus presenting a drug-resistant phenotype. The immobility time scores in Creb1^DBHCre^Crem−/− mice were similar to or greater than those of untreated control littermates; these scores were significantly different from those of single Creb1^DBHCre^ mutants. This phenomenon was observed in Creb1^DBHCre^Crem−/− mice regardless of sex.

### Antidepressant-like effects of fluoxetine in Creb1^TPH2CreERT2^Crem−/− double mutants on the TST are sex-dependent

**Figure 6:**
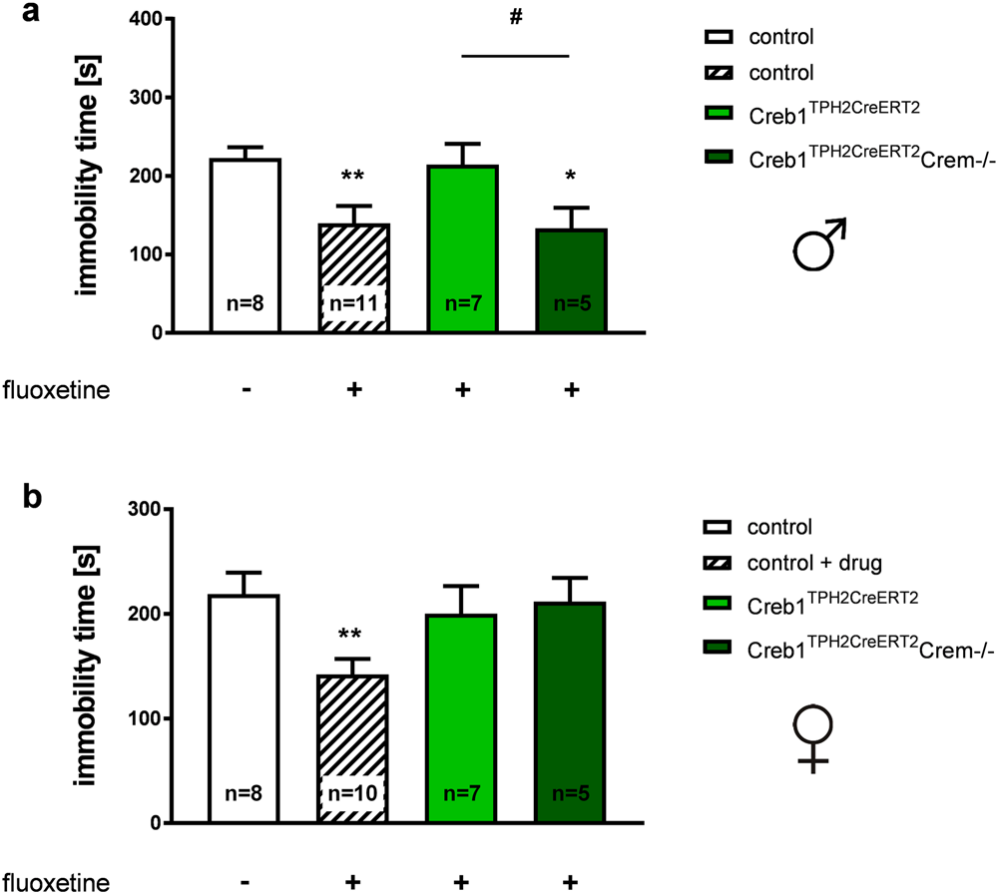
The effect of Creb1 deletion on mouse responsiveness on the TST after fluoxetine. (**a**) Immobility of control, Creb1^TPH2CreERT2^, and Creb1^TPH2CreERT2^/Crem−/− male mice on the TST after fluoxetine treatment (single dose, 10 mg/kg, i.p., 30 min. prior to the test) (ANOVA: F_(3,27)_ = 4.36 p < 0.05; LSD post-hoc test: *p < 0.05, **p < 0.01 vs control; ^#^p < 0.05 vs Creb1^TPH2CreERT2^). (**b**) Immobility of control, Creb1^TPH2CreERT2^, and Creb1^TPH2CreERT2^/Crem−/− female mice on the TST after fluoxetine treatment (single dose, 10 mg/kg, i.p., 30 min prior to the test) (ANOVA: F_(3,26)_ = 3.40 p < 0.05; LSD post-hoc test **p < 0.01 vs control). Data are the mean ± SEM, n = 5–10.

Fluoxetine administration in control male and female mice resulted in significantly shorter immobility times on the TST in comparison to animals that received saline injections (Fig. 6a,b). However, in contrast to the effects observed in Creb1^DBHCre^ and Creb1^DBHCre^Crem−/− mice after desipramine treatment, a single CREB ablation with conserved CREM (Creb1^TPH2CreERT2^ mice) was sufficient to produce a drug-resistant phenotype after fluoxetine administration. The immobility time after fluoxetine injection did not change in these single mutants when compared to control littermates that did not receive the drug. Additionally, when the CREM protein was removed, animals reacted in a sex-dependent manner: Creb1^TPH2CreERT2^Crem−/− double mutant females did not react to fluoxetine treatment, while Creb1^TPH2CreERT2^Crem−/− double mutant males responded to fluoxetine similarly to control animals, exhibiting significantly decreased immobility times relative to saline-treated controls (Fig. 6a,b). These results suggest that cell-specific loss of CREB-dependent signaling could account for differential responses to anti-depressants.

## Discussion

The current study was based on transgenic lines lacking CREB in two important neurotransmitter systems, noradrenergic and serotonergic neurons, both of which play a crucial role in the modulation of antidepressant drug action. The objective of this study was to re-evaluate the role of CREB in antidepressant drug action by considering the known compensatory effects of CREM. This factor has been often ignored in previous investigations of the role of CREB in depression and antidepressant treatment carried out in transgenic animal models. To avoid the compensatory effects of CREM, both lines were maintained in a CREM deficient (Crem−/−) background.

The specificity of the targeted mutation in single mutant Creb1^DBHCre^ line has been previously validated in the central nervous system^35^. The current study confirmed this specificity in the double mutant line Creb1^DBHCre^/Crem−/− as well as in the newly created Creb1^TPH2CreERT2^/Crem−/− double mutant mice. In both cases, the selectivity of the mutation was confirmed by immunofluorescent staining. CREB expression was selectively and completely lost in regions expressing DBH and TPH2: the locus coeruleus (LC; Creb1^DBHCre^/Crem−/− mice; Fig. 1a) and the dorsal raphe nucleus (DR; Creb1^TPH2CreERT2^/Crem−/−; Fig. 1b). Other brain structures remained intact (e.g., hippocampus; Fig. 2a,b).

We quantified the basic phenotype of both transgenic lines in selected behavioral tests. When compared to control littermates, single CREB mutants (Creb1^DBHCre^, Creb1^TPHCreERT2^) and double mutants (Creb1^DBHCre^/Crem−/−, Creb1^TPH2CreERT2^/Crem−/−) displayed no obvious alterations in daily cage behavior, weight gain (Figs 3a,b and 4a,b), spontaneous locomotor activity (Figs 3 c,d and 4c,d), or motor coordination (Figs 3 e,f and 4e,f), regardless of genotype and sex. Since the main purpose of this study was to investigate the influence of select antidepressants on the tail suspension test (TST), basal depressive behavior was evaluated beforehand to ensure that results were not biased by reactiveness on the part of non-treated animals on the TST (Figs 3 g,h and 4g,h). Male and female animals were investigated separately because sex differences frequently occur in studies of depression-like and antidepressant behavior^36^, an issue often neglected by researchers. TST in basal conditions did not reveal any differences between mutant and control animals in both studied transgenic lines. Only difference noted in basal phenotype of studied animals was a diminished locomotor activity of Creb1^DBHCre^ single, and Creb1^DBHCre^ Crem−/− double male mutants in the first 10 min interval of OFT. This might suggest increased anxiety behavior of male mutant mice, but even assuming such phenotype, it does not reflect the results obtained in TST at basal conditions (Fig. 3g). Overall, the only noticeable difference in basal phenotype between mutants and controls was the infertility of CREM-deficient male mice, a well-known issue due to the crucial role of CREM in spermatogenesis^37^.

Abovementioned basal phenotype results indicated that both transgenic models could be evaluated on the TST following antidepressant treatment without risk of confounds due to prior behavioral impairments. We selected a single-dose paradigm using the most common, representative antidepressants: a potent noradrenaline reuptake inhibitor (desipramine) and selective serotonin reuptake inhibotor (SSRI; fluoxetine). Doses (20 mg/kg, i.p. and 10 mg/kg, i.p., respectively) were based on our previous experience and published literature^38,39^.

In the transgenic line targeting the noradrenergic system, neither male nor female Creb1^DBHCre^/Crem−/− mice responded to acute desipramine treatment on the TST. This result differed from findings in single Creb1^DBHCre^ mutants and was consistent with the initial hypothesis that CREM-dependent compensation for CREB function plays a role in antidepressant treatment. Apparently a single mutation was insufficient for disordering the mechanism of drug action, and the effects of desipramine on the TST were abolished only after concomitant CREM removal. This finding is also in line with other results highlighting the pivotal role of CREB in the action of antidepressant drugs targeting the noradrenergic system, in particular desipramine. Specifically, it has been proposed that the therapeutically relevant action of this drug may be related to attenuation of CREB-mediated gene transcription^22^. Recent studies have shown that desipramine improves depression-like behavior on the TST by upregulating p-CREB in the hippocampus^40^. Thus, the functional loss of CREB in Creb1^DBHCre^/Crem−/– mice potentially interferes with these proposed mechanisms.

Interestingly, we did not observe analogous effects in the second line targeting the serotonergic system after fluoxetine application. Regardless of sex, single-mutant Creb1^TPH2Cre^ mice displayed resistance to fluoxetine treatment on the TST. Moreover, this effect was sustained only in case of Creb1^TPH2CreERT2^/Crem−/− female double mutants; in contrast, Creb1^TPH2CreERT2^/Crem−/− male double mutants responded to fluoxetine treatment in the same way as control mice. One of possible explanation that mires interpretation of obtained results might be the fact, that due to technical limitations and mice availability, in our experiments we compare constitutive (Creb1^TPH2CreERT2^/Crem−/−) vs inducible (Creb1^TPH2CreERT2^/Crem−/−) line. Therefore, before introducing drug factor, we thoroughly analyzed the basic behavior of both transgenic lines and did not find any discerning effects of introduced mutation in neither of them. Nevertheless, one cannot exclude that compensatory effects may emerge when triggered by external stimuli i.e. exposure to the drug. We speculate that in case of Creb1^TPH2CreERT2^ mice, CREM-dependent brain plasticity was insufficient to compensate for the single CREB ablation especially because the mutation took place in adult mice which eliminates the possible developmental compensatory mechanisms present in constitutive knock-out animals. This assumption is supported by prior findings that CREM overexpression in transgenic models is sometimes insufficient to prevent the CREB-deletion phenotype, particularly in the context of drug addiction^31^. Moreover, the precise mechanisms of interaction between CREB, CREM and ATF factors (including the extent and kinetics of mCREB dimerization) are not well understood^37,41^. On the other hand, the response to fluoxetine treatment observed in Creb1^TPH2CreERT2^/Crem−/− male mice could be interfered by different mechanism of action of drugs acting via the serotonergic system, particularly fluoxetine, whose effectiveness is additionally determined by various environmental factors^42^. This finding is compatible with observations of differential regulation of CREB expression by desipramine and fluoxetine and with the regional specificity of their effects^43^.

In relation to the gender-dependent response to fluoxetine observed in serotonergic-specific mutants, interestingly, clinical studies have reported sex differences among prevalence of depression, particularly among perimenopausal women^44^. Differential responsiveness to antidepressants, including SSRIs, has been reported between men and women in clinics, although this finding remains controversial^45^. Furthermore, sex differences in the behavioral responsiveness of transgenic mouse models targeting the serotonergic system have been described in other studies as well^34,46,47^.

Finally, it has to be clearly stated that all previous studies regarding the role of CREB in depression and antidepressant drug action have been carried out in multiple brain regions, while our mutation is focused on the origin sites of noradrenergic and serotonergic neurons in the central nervous system: in particular, the locus coeruleus (LC) and dorsal raphe (DR) nuclei. It remains uncertain whether and how CREB can influence other brain structures receiving inputs from noradrenergic and/or serotonergic projections. These structures are traditionally regarded as the most important areas in depression pathophysiology and the mechanism of antidepressants (i.e., prefrontal cortex, hippocampus, nucleus accumbens). Clarifying these influences will be our goal in future research. Due to technical limitations (i.e., the demands of obtaining proper groups of controls, single mutants, and double mutant littermates for behavioral experiments, which forced us to split the analysis of basal and drug-induced behavior in mutant mice), we were unable to include a separate group of single-mutation CREM-deficient mice (Crem−/−). Crem−/− male mutants are known to be sterile^48^, but overall this mutation does not appear to be phenotypically meaningful, being not associated with any profound impairment^49^. Namely, no significant difference were observed in daily cage behavior, spontaneous and amphetamine-induced locomotor activity, conditioned suppression of motility (reactiveness to stress)^49^. Crem−/− mice were characterized as slightly hyperactive only when analyzed in the dark phase, and by diminished anxiety behavior as revealed by elevated plus maze (EPM)^49^. However, this phenotype (even assuming exertion on obtained results) should in fact promote antidepressant behavior of double mutants analyzed in our experiments i.e. decreased immobility in TST at basal conditions, and such behavior was certainly not observed.

These initial observations, in particular those regarding the effects of fluoxetine, require confirmation in other behavioral paradigms. Nevertheless, these results provide overall confirmation of the crucial role of CREB in response to antidepressant treatment and clearly highlight CREM as an important compensatory factor, despite the different regulation observed in the serotonergic line. Additionally, these newly-created models based on functional ablation of CREB in select neuronal populations may represent a unique, valuable tool for investigating the role of CREB in the mechanism of antidepressants.
